# Gut Microbiota Modulation as a Potential Target for the Treatment of Lung Infections

**DOI:** 10.3389/fphar.2021.724033

**Published:** 2021-09-07

**Authors:** Clênio Silva Cruz, Mayra Fernanda Ricci, Angélica Thomaz Vieira

**Affiliations:** Laboratory of Microbiota and Immunomodulation (LMI), Department of Biochemistry and Immunology, Institute of Biological Sciences, Federal University of Minas Gerais, Belo Horizonte, Brazil

**Keywords:** symbiotics, mucosal immmunity, gut-lung axis, prebiotcs, probiotics, immunobiotics, inflammation, microbiota

## Abstract

The gastrointestinal and respiratory systems are colonized by a complex ecosystem of microorganisms called the microbiota. These microorganisms co-evolved over millions of years with the host, creating a symbiotic relationship that is fundamental for promoting host homeostasis by producing bioactive metabolites and antimicrobial molecules, and regulating the immune and inflammatory responses. Imbalance in the abundance, diversity, and function of the gut microbiota (known as dysbiosis) have been shown to increase host susceptibility to infections in the lungs, suggesting crosstalk between these organs. This crosstalk is now referred to as the gut-lung axis. Hence, the use of probiotics, prebiotics, and synbiotics for modulation of gut microbiota has been studied based on their effectiveness in reducing the duration and severity of respiratory tract infections, mainly owing to their effects on preventing pathogen colonization and modulating the immune system. This review discusses the role and responses of probiotics, prebiotics, and synbiotics in the gut-lung axis in the face of lung infections.

## Introduction

Microorganisms and humans have co-evolved for thousands of years, and many survival functions have been defined throughout this time for both. All body surfaces are colonized by complex and dynamic communities of symbiotic microorganisms, including bacteria, viruses, fungi, helminths, and protists, called microbiota (Grice and Segre, 2012; Sender et al., 2016). As demonstrated by next-generation sequencing, the lungs and gut possess unique microbiota that differ mainly in composition and structure, with bacteria being the most predominant microorganisms (Dickson et al., 2015; Santacroce et al., 2020). The microbiota plays fundamental roles in host homeostasis via the metabolism of nutrients, production of vitamins, metabolites, and antimicrobial molecules, activation of the immune system, and regulation of the inflammatory process (Dang and Marsland, 2019). The gut dysbiosis has been shown to increase susceptibility to infection in the lungs, and infections in the lung are identified as a cause of gut dysbiosis; highlighting a bidirectional link between these two organs; this crosstalk is now called the gut-lung axis (Li et al., 2008; Hand et al., 2016; Budden et al., 2017; Sencio et al., 2020). Also, the lung and gut originate from the same embryonic organ, the foregut, and consequently have some structural similarities that might contribute to the interaction between these two organs (Faure and de Santa Barbara, 2011). Respiratory tract infections (RTI) are a global health concern. Approximately 2.38 million deaths were attributed to RTI in 2016 alone, making it the sixth leading cause of mortality among all ages and the leading cause of death among children under 5 years (Ferkol and Schraufnagel, 2014; Troeger et al., 2018). Given the importance of the gut-lung axis, our review summarized the latest experimental and clinical studies on this topic and showed that modulation of the gut-lung axis with probiotics, prebiotics, and synbiotics, could be an important therapeutic target for preventing and treating lung infections caused by bacteria, viruses, fungi, and parasites.

## Gut-Lung Axis in Respiratory Tract Infections

**FIGURE 1:**
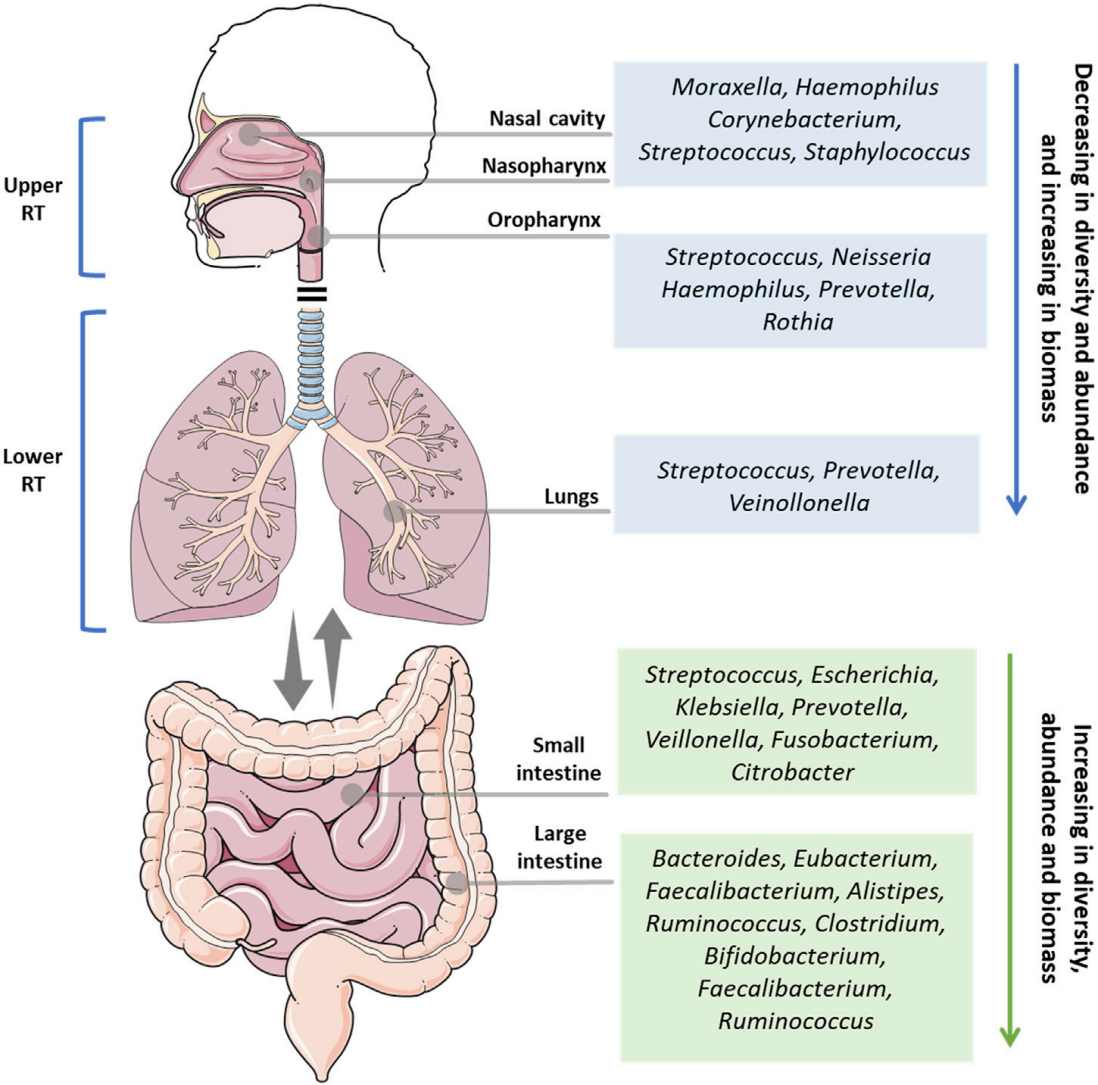
Overview of the main microbial genus in the health upper respiratory tract (nasal cavity, nasopharynx, oropharynx), lower respiratory tract (lungs), small intestine, and large intestine. RT: respiratory tract.

The respiratory system is composed of different organs, and is divided into two main parts: the upper respiratory tract (URT) and the lower respiratory tract (LRT). The URT comprises the nostrils, nasal passages, paranasal sinuses, nasopharynx, and oropharynx, while the lower respiratory tract comprises the trachea, bronchi, bronchioles, and alveoli. These organs make up one of the largest surface areas in the human body, that from the nostrils to the lungs, is colonized by a symbiotic and diverse community of microorganisms (Figure 1).

The microbiota of the lungs and gut of healthy individuals differ significantly in taxonomic composition, diversity, and function. In contrast to the thriving resident microbiota in the gut, the lung microbiota is composed of transient microorganisms mainly derived from URT. While Bacteroidetes and Firmicutes are the most abundant bacterial phyla in both microbiotas, the lung and gut microbiota are very different at the species level. In the lungs, the genera *Streptococcus* spp., *Veillonella* spp., and *Prevotella* were the most abundant, whereas *Bacteroides*, *Faecalibacterium*, and *Bifidobacterium* are more prevalent in the gut (Sender et al., 2016). In a disease or dysbiotic state, other organisms are present in the lung, such as viruses, including human rhinovirus, human bocavirus, polyomaviruses, human adenovirus, and human coronavirus, and fungi such as *Aspergillus* spp., *Penicillium* spp., *Candida* spp., and *Alternaria* spp. (Papadopoulos and Skevaki, 2006; Limon et al., 2017).

The immune responses in the gut-lung axis depend on the balance of microbiota composition, particularly in the gut. The regulated interaction between the metabolites and antigens of symbiotic microbiota with the host is crucial for the activation of pattern recognition receptors (PRRs) and metabolic sensor receptors such as G-protein-coupled receptors (GPCRs), and the production of inflammatory mediators, which are necessary for the migration, activation, and proliferation of innate and adaptive immune cells responsible for the production of pro-and anti-inflammatory cytokines, immunoglobulins, and antimicrobial peptides (Fan and Pedersen, 2021). These cells and molecules can move bidirectionally between the lungs and the gut through the bloodstream and lymphatic system and regulate immune and inflammatory responses (Marsland et al., 2015; Dang and Marsland, 2019).

Intestinal dysbiosis is responsible for increasing the susceptibility of the host to lung disease, as evidenced by the high prevalence of asthma in patients with irritable bowel syndrome (Yazar et al., 2001). Experimentally, mice treated with antibiotics are more susceptible to lethal infection by the influenza virus (IFV) (Ichinohe et al., 2011; Pang et al., 2018). Furthermore, infections in the lungs are also linked to dysbiosis in the gut; mice infected with IFV displayed a significant increase in *Enterobacteriaceae* and decreased diversity of *Lactobacillus* and *Lactococcus* (Wang et al., 2018). Influenza infection also affects the production of short-chain fatty acids (SCFAs) and impairs the gut barrier properties thereby increasing susceptibility to second bacterial infections (Sencio et al., 2020, 2021).

SCFAs, such as butyrate, propionate, and acetate derived from the fermentation of dietary fibers by the microbiota, are involved in regulating the inflammatory process and pulmonary immune response (Fukuda et al., 2011; Trompette et al., 2014). SCFAs activate GPCRs and inhibit histone deacetylases, thus contributing to the reduction of inflammation in the gut-lung axis by inhibiting the NF-κB signaling pathway, increasing regulatory T (Treg) cells, and decreasing T helper 1 (Th1) and Th17 cells (Maslowski et al., 2009; Kim et al., 2013; Li et al., 2018). SCFAs can also reach the bone marrow and influence the generation and development of immune cells such as Ly6C- and Ly6C + monocytes and dendritic cells, which can be recruited into the lungs and modulate the immune response against pathogens (Trompette et al., 2014, 2018; Kopf et al., 2015). Our research group has also demonstrated that activation of the GPR43 receptor in neutrophils and alveolar macrophages by acetate is essential for modulating the inflammatory response and controlling pulmonary infection by *Klebsiella pneumoniae* (Galvão et al., 2018) and *Streptococcus pneumoniae* serotype 1 in mice (Sencio et al., 2020). In another study, activation of GPR43 in pulmonary epithelial cells induced interferon (IFN)-β in the lungs and increased the protection of mice infected with respiratory syncytial virus (RSV) (Antunes et al., 2019).

## Probiotics, Prebiotics, and Synbiotics

**FIGURE 2:**
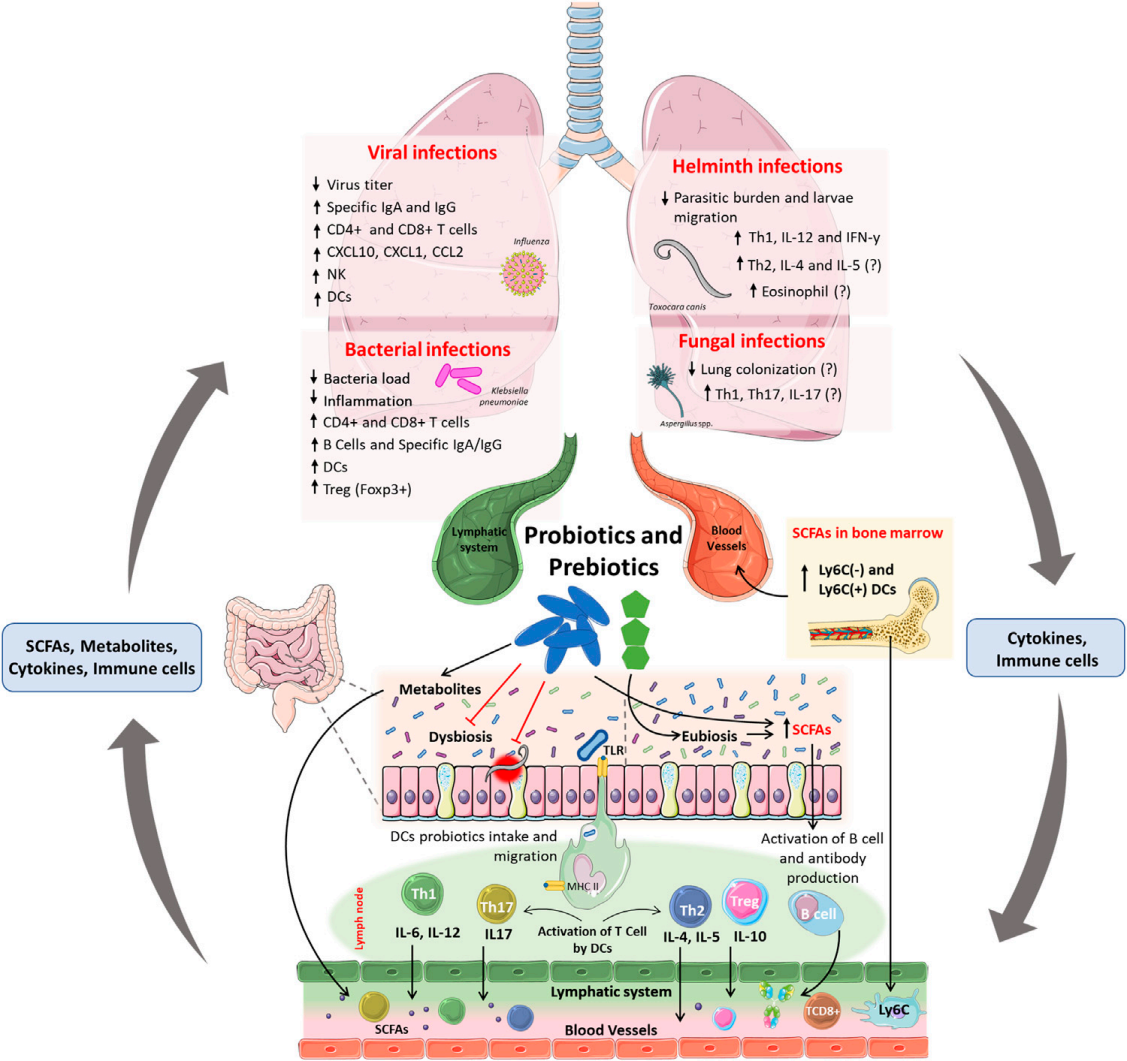
Effects and mainly mechanisms of probiotics and prebiotics in the gut-lung axis and context of respiratory infections. Probiotics and prebiotics administered orally can improve dysbiosis and induce eubiosis in the host, leading to an increase in SCFAs directly (produced by probiotics) or indirectly (produced by commensal microbiota). Furthermore, probiotics can also reduce the burden and epithelial damage induced by intestinal parasites. The uptake of probiotics by DCs in the intestinal submucosa, and their migration to lymph nodes, induces the activation and proliferation of Th1, Th2, Th17, Treg, and B cells. Activated T cells and B cells produce cytokines and antibodies, enter the circulatory and lymphatic systems, and reach the lungs, where they will increase resistance to infections caused by viruses, bacteria, and fungi. The fermentation of prebiotics and production of SCFAs increases the number of DCs precursors in the bone marrow and increases CD8^+^ T cells activity, that confer protection against infections in the lung. The immunomodulation demonstrated after the administration of probiotics and prebiotics may be linked to the reduced viral titer, bacterial colonization, parasite load, and migration in the lungs. Probiotic-induced immunomodulation can increase the frequency of dendritic cells and CD4^+^ and CD8^+^ T cells in the lungs against infections by viruses and bacteria and can increase specific IgG and IgM antibodies to these pathogens. Also, the increase in Treg cells may be related to the reduction of inflammation-induced lung damage. In parasitic infections, probiotics have been linked with increased frequency of Th1 and concentration of IL-12 and IFN-γ, which may justify the reduction in the parasite load and larvae migration in the lung. Because there are no scientific studies that demonstrate the reduction of lung colonization by fungi after oral administration of probiotics, is still unknow if the antimycotic potential from probiotics metabolites, as shown *in vitro*, could be applied in an *in vivo* system.

Probiotics are live microorganisms that confer benefits to the host when administered in adequate amounts (Salminen et al., 2021). Probiotics are considered important tools for the modulation of microbiota in the gut-lung axis, with their benefits on the gut-lung axis dependent on the strains used (Figure 2). However, common mechanisms have been reported between species, such as –1) colonization of the respiratory and intestinal tracts, 2) production of SCFAs and antimicrobial peptides, 3) maintenance of the integrity of the intestinal and pulmonary mucosa, and –4) stimulation of the innate and adaptive immune system (Bermudez-Brito et al., 2012; Salminen et al., 2021). The benefits of probiotics have been shown in animal models and clinical studies in many disease conditions, such as post-antibiotic-associated diarrhea, allergies and inflammatory bowel diseases, and respiratory tract infections (Vieira et al., 2013). For a given microorganism to be assessed as a probiotic, biosafety criteria and scientific evidence regarding its biological benefits must be considered (Harzallah and Belhadj, 2013). *Lactobacillus* and *Bifidobacterium* species are more commonly used as probiotics; however, yeasts, certain *Streptococcus* spp. strains, and *Bacillus* spp. are also used as probiotics, but less frequently (Fijan, 2014). The use of inactivated probiotics is also of great interest because live probiotic microorganisms may cause systemic infections, excessive immune stimulation, and antibiotic resistance gene transfer (Doron and Snydman, 2015). Taking this into consideration, the term postbiotics was proposed as preparation for inanimate microorganisms and/or their components that confer a health benefit on the host (Salminen et al., 2021).

Prebiotics are dietary fibers, such as inulin, fructooligosaccharides, and galactooligosaccharides, which are fermented in the gut and promote an increase in the diversity and activity of specific symbiotic microorganisms (Salminen et al., 2021). The activity of prebiotics also leads to an enhancement of immune response, decrease in colon pH, local induction of reactive oxygen species (ROS), trophic effects on enterocytes, and anti-inflammatory responses (Vieira et al., 2013). In addition, the SCFAs butyrate and propionate, derived from the metabolism of prebiotics, can increase miRNAs through the inhibition of histone deacetylases, leading to improved antibody class switching and local and systematic impact on the T-dependent and T-independent immunoglobulin production (Sanchez et al., 2020).

Synbiotics consist of probiotics and prebiotics to achieve synergistic and complementary effects on their functions (Salminen et al., 2021). A recent meta-analysis of randomized controlled clinical trials involving over 10,000 individuals showed the effectiveness of synbiotic interventions in reducing the rate of respiratory tract infections (Chan et al., 2021). Understanding the specific mechanisms of interaction between probiotics and prebiotics and their modulation of the gut environment and immune response will lead to better utilization of the synbiotics to treat infections and metabolic diseases.

### Gut-Lung Axis Modulation in the Context of Bacterial Lung Infections

The lung is highly vulnerable to bacterial infections due its constant exposure to environment agents. One of the most common diseases caused by bacteria in the lungs is pneumonia that is characterized by alveolar infection and intense inflammatory response that ranges from mild to severe and can affect both the right and left lobes and may impair the gaseous exchange. The most common causes of bacterial pneumonia in immunocompetent hosts include *S. pneumoniae*, *Haemophilus* spp., and *Mycobacterium tuberculosis.* In immunocompromised hosts the number of pathogens that cause pneumonia is much larger, and in general those individuals are more vulnerable and have worse outcomes (van der Poll and Opal, 2009) (Table 1).

**TABLE 1:** Pre-clinical studies on the modulation of the microbiota for treatment of bacterial and viral lung infections.

Strategy for microbiota modulation	Dose and route of administration	Experimental model	Pathogen	Main outcomes	References
Effects on bacterial pathogen					
*Lactobacillus bulgaricus* CRL 423 and *Streptococcus thermophilus* CRL 412	2 × 10^8^ CFU, via oral	Malnourished, Swiss albino mice	*Streptococcus pneumoniae*	Reduced bacterial load in the lungs; increased bactericidal function of bronco-alveolar phagocytes; reduced tissue inflammation; increased neutrophils in blood; and increased level of lung anti-pneumococcal IgA and IgG	Villena et al. (2006)
*Lactobacillus casei* CRL 431	1 × 10^9^ CFU, via intranasal	Malnourished, Male, 3-week-old Swiss albino mice	*Streptococcus pneumoniae*	Increased the bacteria lung clearance; improved production of TNF-α; increased activity of phagocytes in the respiratory tract; increased IL-4, IL-10, and *Pneumococcus-*specific IgG	Villena et al. (2009)
*Lactobacillus casei* CRL 431	1 × 10^9^ CFU, via oral	Male 6-week-old Swiss albino	*Streptococcus pneumoniae*	Increased pathogen clearance from blood; lower lung damage; improved number of leukocytes and neutrophils; and increased levels of antipneumococcic IgA in BAL.	Villena et al. (2005)
*Lactobacillus fermentum*	1 × 10^7^, via intranasal	Adult, BALB/c mice	*Streptococcus pneumoniae*	Increased the number of activated macrophages and lymphocytes; and increased anti-*S.pneumoniae* antibodies	Cangemi De Gutierrez et al. (2001)
*Lactobacillus rhamnosus* CRL1505	1 × 10^8^ CFU, via intranasal	Malnourished, Male, 3-week-old Swiss-albino mice	*Streptococcus pneumoniae*	Changed the quantitative and qualitative alterations of CD4^+^ T cells in the bone marrow, thymus, spleen and lung induced by malnutrition and infection; and increased IL-10 and IL-4 in respiratory and systemic compartments	Barbieri et al. (2017)
*Lactobacillus casei* CRL 431, *Lactobacillus delbrueckii* subsp*. bulgaricus* and *Streptococcus thermophilus*	1 × 10^9^ CFU, via oral	3-week-old, Swiss albino mice	*Pseudomonas aeruginosa*	Enhanced lung clearance of *P. aeruginosa*; increased phagocytic activity of alveolar macrophages; and increased IgA and IgM levels in BAL.	Alvarez et al. (2001)
*Lactobacillus rhamnosus* GG	4 × 10^8^ CFU, via oral	5 to 8-week-old, FVB/N mice	*Pseudomonas aeruginosa*	Mice treated had improved survival; reduced bacterial counts in BAL; decreased the levels of IL-6 and increased levels of IL-10 mRNA; improved lung pathology; and increased levels of Treg cell marker Foxp3	Meitert et al. (2013)
*Lactobacillus fermentum* K.C6.3.1E, *Lactobacillus zeae* Od.76, and *Lactobacillus paracasei* ES.D.88	9 × 10^6^ CFU, via intratracheal	6 to 8-week-old, C57BL/6 mice	*Pseudomonas aeruginosa*	Decreased secretion in BAL of IL-6 and TNF-α	Fangous et al. (2019)
*Bifidobacterium longum* 5^1A^	1 × 10^8^, via oral	8 to 12-week-old, C57BL/6 WT and Mal/TIRAP^-/-^ mice	*Klebsiella pneumoniae*	Reduced bacterial burden; faster resolution of inflammation; decreased lung damage; increased production of IL-10; and increased alveolar macrophages ROS associated with Mal/TIRAP activation	Vieira et al. (2016)
Effects on viral pathogen					
*Lactobacillus casei* Shirota	1 × 10^8^ CFU, via oral	Neonatal and infant, BALB/c mice	Influenza A/PR/8/34 (H1N1)	Higher survival rate, reduced titer of virus in the nasal washings; greater pulmonary NK cell activity; and increased IL-12 production by mediastinal lymph nodes	Yasui et al. (2004)
*Lactobacillus rhamnosus* M21	1 × 10^9^ CFU, via oral	Female, specific pathogen-free, BALB/c mice	Influenza A/NWS/33 (H1N1)	Increased IL-2 and IFN-γ; increased sIgA levels; reduced inflammatory cells in BAL.	Song et al. (2016)
*Lactobacillus pentosus* S-PT84	Heat-killed *L. pentosus* S-PT84, via intranasal	Female, BALB/c mice	Influenza A/PR/8/34 (H1N1)	Increased survival rates; reduced titer of influenza virus in BAL; increased IL-12 and IFNγ production in mediastinal lymph node cells; increased IL-12 and IFN-α in BAL; and increased NK cell activity	Izumo et al. (2010)
*Lactobacillus plantarum* and *Leuconostoc mesenteroides*	1 × 10^9^ CFU, via oral	Female, 5-week-old, BALB/c mice	Influenza rK09 (H1N1)	Restrained viral replication; and increased rates of survival of infected mice	Bae et al. (2018)
*Lactobacillus rhamnosus* GG	200 µg of *L. rhamnosus* GG lyophilized, via intranasal	Female, 7-week-old BALB/c mice	Influenza A⁄PR⁄8⁄34 (H1N1)	Higher survival rate; increased cell-killing activity of lung cells; and increased mRNA expression of interleukin IL-1 beta, TNF and MCP-1	Harata et al. (2010)
*Lactobacillus acidophilus* L-92	4 × 10^10^ CFU, via oral	Female, 4-weeks-old BALB/c mice	Influenza A/PR/8/34 (H1N1)	Reduced Virus titers in the lung; increased NK cells activity; decreased the number of neutrophils; increased eotaxin, MCSF, IL-1β, RANTES and IFN-α in the lung; and increased IL-17 levels in Peyer’s patches	Goto et al. (2013)
*Lactobacillus plantarum* DK119	1 × 10^8^ or 1 × 10^9^ CFU, via oral	Female, BALB/c mice	Influenza A/PR8	Reduced lung viral loads; increased levels of cytokines IL-12 and IFN-γ in BAL; and reduced degree of inflammation	Park et al. (2013)
*Lactococcus lactis* subsp. *lactis* JCM5805	1 mg of heat-killed *L. lactis* subsp. *lactis* JCM5805, via oral	Female, DBA/2jjcl mice	Murine Parainfluenza virus (mPIV1)	Increased survival rate; prevention of weight loss; reduced lung histopathology scores; increased activation of Peyer’s patches (PP) and PP pDCs; increased levels of type I IFNs; and increased expressions of anti-viral factors such as *Isg15*, *Oasl2*, and *Viperin*, at lung	Jounai et al. (2015)
*Lactobacillus paracasei* CNCM I-1518	2 × 10^8^ CFU, via oral	Female, 6-week-old BALB/c mice	Influenza A/Scotland/20/74 (H3N2)	Reduced weight loss; and increased recruitment of inflammatory myeloid cells, such as interstitial monocytes and dendritic cells, to the lungs	Belkacem et al. (2017)
*Bacillus subtilis* 3 (UCM B-5007)	1 × 10^7^ CFU, via oral	Four-week-old BALB/c mice	Influenza A/FM/1/47 (H1N1)	Prevented influenza infection	Starosila et al. (2017)
*Lactobacillus rhamnosus CRL1505*	1 × 10^8^ CFU, via oral	Male, 6-week-old BALB/c mice	Respiratory Syncytial Virus strain A2 and Influenza virus A/PR/8/34 (H1N1)	Reduced lung immune-coagulative reaction triggered by TLR3 activation	Zelaya et al. (2014)
*Lactobacillus plantarum* NCIMB 8826 and *Lactobacillus reuteri* F275	1 × 10^9^ CFU, via intranasal	BALB/c and C57BL/6 MyD88^−/−^ mice	Pneumonia Virus of mice (PVM) strain J3666	Protection against lethal infection; reduced granulocyte recruitment; reduced expression of proinflammatory cytokines CXCL10, CXCL1, CCL2, and TNF.	Gabryszewski et al. (2011)
*Lactobacillus rhamnosus* CRL1505 and CRL1506	1 × 10^8^ CFU, via intranasal	Female, 3-week-old BALB/c mice	Human RSV strain A	Increased levels of IFN-α, IFN-β, IFN-γ, IL-6 and IL-10; increased levels of CD4^+^ Tregg cells and CD11c^+^CD103^+^ DCs; reduced viral replication and lung damage	Tomosada et al. (2013)

Several studies have shown that the oral administration of different strains of probiotics, such as *Lactobacillus bulgaricus* CRL 423 and *Streptococcus thermophilus* CRL 412 (Villena et al., 2006), *L. casei* CRL 431 (Villena et al., 2005, 2009), *L. fermentum* (Cangemi De Gutierrez et al., 2001), and *L. rhamnosus* CRL 1505 (Barbieri et al., 2017) causes: 1) increased resistance to infection, 2) decreased number of bacteria in the lungs, and 3) increased survival of mice infected with *S. pneumoniae*. In general, these articles, associated this protection with an increase in neutrophils, lymphocytes, macrophages, phagocytic activity, and levels specific anti-*S. pneumoniae* IgG and IgA in the lungs. The increase in phagocytic activity and the number of neutrophils in the lower respiratory tract is the first line of defense against invading pathogens, and the increase in regulatory cells and cytokines contributes to the reduction of the inflammatory response and to the maintenance of tolerance to symbiotic microorganisms, which is necessary to reduce damage associated with infections by pathogens (Martin and Frevert, 2005).

Similar results were observed in mice infected with *Pseudomonas aeruginosa*, treated orally with the probiotics *L. casei* CRL 431, *L. delbrueckii* subsp. *bulgaricus*, *S. thermophilus* (Alvarez et al., 2001) and *L. rhamnosus* GG (Meitert et al., 2013). In addition, the administration of *L. rhamnosus* GG induces an anti-inflammatory response by increasing the levels of regulatory T cells (Treg) Foxp3+ and decreasing the production of the proinflammatory cytokine IL-6. This anti-inflammatory profile was also observed in mice infected with *P. aeruginosa* with intratracheal administration of probiotics *L. fermentum* K.C6.3.1E, *L. zeae* Od.76, and *L. paracasei* ES.D.88, demonstrated by the reduction of lung inflammation and decreased production of IL-6 and tumor necrosis factor (TNF)-α (Fangous et al., 2019).

In an experimental lung infection by *K. pneumoniae*, the administration of viable or inactivated probiotic *Bifidobacterium longum* 5^1A^ induced pulmonary clearance of *K. pneumoniae* by increasing ROS production in alveolar macrophages through activation of the mal/TIRAP signaling pathway (Vieira et al., 2016). There was a concomitant reduction in the inflammatory process and concentration of cytokines TNF-α and IL-6 and an increase in IL-10 in the lungs of mice. However, only viable probiotics were able to increase the levels of IL-10 in the lungs of mice. Viable *B. longum* 5^1A^ produces SCFA acetate in large quantities, and acetate administration in mice before respiratory infection by *K. pneumoniae* induced increased production of IL-10 in animals. The authors demonstrated that acetate might be the primary inducer production of IL-10 in this model (Vieira et al., 2016). In addition, intestinal colonization of germ-free mice with *B. longum* 5^1A^ restored the ability of these mice to decrease infection by increasing the production of CXCL1 and the recruitment of neutrophils (Vieira et al., 2016). In mice infected with IFV, the lower quantity of acetate was also related to increased susceptibility to secondary respiratory pneumococcal infection, mainly due to the impaired bactericidal activity of alveolar macrophages, and this detrimental effect was restored after acetate supplementation (Sencio et al., 2020).

The potential of probiotics to protect the host from pulmonary infections has also been assessed in several clinical studies, including diverse patients, methodological designs, and inclusion criteria (Table 2). Most of these studies focused on probiotics for prevention and treatment of nosocomial pulmonary infections in patients admitted to intensive care units (ICUs). Two prospective, randomized, double-blind, and placebo-controlled studies showed that the probiotics *L. casei rhamnosus* Lcr35 and *L. rhamnosus* GG, administered orally or oropharyngeally, resulted in decreased colonization and infection of the LRT by *P. aeruginosa* or related gram-positive and gram-negative pathogens in patients admitted to the ICU using mechanized pulmonary ventilation (Forestier et al., 2008; Morrow et al., 2010). Only one study showed that administration of a synbiotic consisting of *B. breve* Yakult, *L. casei* Shirota, and galactooligosaccharides decreased the incidence of ventilator-associated pneumonia (VAP) in patients diagnosed with sepsis admitted to the ICU (Shimizu et al., 2018).

**TABLE 2:** Clinical studies on the modulation of the microbiota for treatment of bacterial and viral lung infections.

Strategy for microbiota modulation	Dose and route of administration	Study design and subjective	Pathogen and disease	Outcome	References
Effects on bacterial pathogen					
Lactobacillus casei rhamnosus Lcr35	1 × 10^9^ CFU, via oral	Prospective, randomized, double-blind, placebo-controlled pilot study with patients aged 18-91	*Pseudomonas aeruginosa*	Reduction in the occurrence of *P. aeruginosa* respiratory colonization and/or infection in the probiotic group. Reduction in the frequency of VAP to *P. aeruginosa*	Forestier et al. (2008)
*Lactobacillus rhamnosus* GG	2 × 10^9^ CFU, via oral	Prospective, randomized, double-blind, placebo-controlled trial with patients at high risk of developing VAP	VAP by Gram-positive and Gram-negative pathogens	Reduction in the development of microbiologically confirmed VAP. Patients treated with probiotics had fewer days of antibiotics prescribed for VAP.	Morrow et al. (2010)
*Bifidobacterium breve* Yakult, *Lactobacillus casei* Shirota, and galacto-oligosaccharide	1 × 10^8^ CFU of *B. breve* and *L. casei* and Galacto-oligosaccharides in a 10 g/day formula, via oral	Randomized controlled trial with patients with more than 16 years old	Patients with more than 16 years old, placed on a ventilator, and who were diagnosed as having sepsis	Reduced incidence of VAP. Increased number of *Bifidobacterium* and *Lactobacillus.* Increased concentration of acetate in the feces	Shimizu et al. (2018)
Effects on viral pathogens					
*Lactobacillus rhamnosus* GG ATCC 5310 and galacto-oligosaccharide and polydextrose mixture	1 × 10^9^ CFU/day for 1–30 days and 2 × 10^9^ CFU/day for 31–60 days, via oral	Randomized, double-blind, placebo-controlled study with preterm infants	Rhinovirus-associated respiratory tract infection	Reduced respiratory tract infections. Reduced rhinovirus-induced episodes	Luoto et al. (2014)
*Lactobacillus brevis KB290*	6 × 10^9^, via oral	Open-label, parallel-group trial with elementary schoolchildren	Influenza infection	Reduced incidence of influenza infections	Waki et al. (2014)
*Lactobacillus rhamnosus GG*	1 × 10^9^, via oral	Randomized, double-blind, placebo-controlled study with nursing home residents aged 65 and older	Influenza infection	Reduced laboratory-confirmed respiratory viral infections	Wang et al. (2018)
*Streptococcus thermophilus DSM 32245, Bifidobacterium lactis DSM 32246, Bifidobacterium lactis DSM 32247, Lactobacillus acidophilus DSM 32241, Lactobacillus helveticus DSM 32242, Lactobacillus paracasei DSM 32243, Lactobacillus plantarum DSM 32244, and Lactobacillus brevis DSM 27961*	2.4 × 10^9^, via oral	Retrospective, observational cohort study with adults	COVID-19 pneumonia by SARS-CoV-2	Increased survival rates of patients that received BAT plus oral bacteriotherapy	Ceccarelli et al. (2021)

In general, we can conclude that the modulation of the intestinal microbiota, mainly with probiotics, is an exciting alternative for treating lung diseases caused by bacteria. Although it is already well established in the literature that probiotic species and strains behave differently according to their metabolic pathways and their interaction with the host, the probiotic species used against lung alterations attract attention to those from the *Lactobacillus* genus. Most articles demonstrated that non-specific immune responses mediated by probiotics, prebiotics, and symbionts are the principal host protection against lung bacteria. Generally, it seemed more significant phagocytic activity of lung macrophages, reduced lung bacterial load, and less tissue inflammation, associated with increased levels of IL-4 and IL-10, increased frequency of Treg cells, increased production of IgA and IgG, and reduced of IL-6 and TNF-α levels. This demonstrates a more resolving anti-inflammatory profile after modulation of the host’s intestinal microbiota. Despite the benefits, these articles use different study designs, experimental models, doses, and routes of administration, making it challenging to translate the results obtained in animal models to humans and thus develop more specific therapies with probiotics.

### Gut-Lung Axis Modulation in the Context of Lung Viral Infections

Viral infections generally cause common cold, bronchiolitis, and pneumonia and vary widely in severity depending on age, immune and nutritional status, genetics, and use of antibiotics. IFV, RSV, and rhinovirus are the most abundant and common causes of lung infections (Jain, 2017). IFV is well known to cause outbreaks of varying severity every year, but recently the novel coronavirus SARS-CoV-2 has emerged as a pandemic that has caused more than 3.5 million deaths (Zhu et al., 2020) (Table 1).

Several studies have demonstrated the potential for oral and intranasal administration of probiotics such as *L. casei* Shirota (Yasui et al., 2004), *L. rhamnosus* M21 (Song et al., 2016), *L. pentosus* S-PT84 (Izumo et al., 2010), and *L. plantarum* and *Leuconostoc mesenteroides* (Bae et al., 2018) to protect and increase the survival of IFV-infected animals, mainly by inducing anti-viral immune responses with the activation of NK cells and increased production of cytokines such as IL-12 and IFN-γ, increased production of IgA in the respiratory mucosa, and reduction of polymorphonuclear inflammatory infiltrate in the lung tissue. In addition to these protective effects, *L. rhamnosus* GG administered intranasally (Harata et al., 2010), and *L. acidophilus* L-92 (Goto et al., 2013) also demonstrated the ability to increase the levels of proinflammatory cytokines, such as IL-1β, monocyte chemotactic protein 1 (MCP-1), and chemokines such as eotaxin and M-CSF.

Dendritic cells are crucial for developing immune responses because of their ability to detect pathogens through TLRs and create a link between innate and adaptive immune responses. Mice with depleted alveolar macrophages lost the anti-viral protection against IFV infection conferred by increasing IL-12 and IFN-γ levels after oral administration of the probiotic *L. plantarum* DK119 (Park et al., 2013). In addition, the importance of dendritic cells was demonstrated after oral administration of the probiotic *Lactococcus lactis* subsp. *lactis* JCM5805 in mice infected with murine parainfluenza virus. The authors showed that the probiotic was incorporated into CD11c^+^ immune cells in Peyer’s patches and activated plasmacytoid dendritic cells that produce type I IFNs at draining mucosal sites. The authors also observed an increase in IFN-related genes, such as *lsg15*, *Oasl2*, and *Viperin* in the lungs, suggesting that the type I IFN produced by plasmacytoid dendritic cells could reach systemic levels and induce anti-viral activity in the lungs. In addition, *ex vivo* stimulation with murine parainfluenza virus of lung lymphocytes from mice treated with JCM5805 demonstrated high expression of IFN-α and IFN-β (Jounai et al., 2015).

Determining the taxonomic composition and function of the microbiota is crucial for understanding the impact of probiotics on the protective response against pathogens. The oral administration of *L. paracasei* CNCM I-1518 did not modify the gut microbiota structure in mice infected with IFV; however, it conferred protection against the virus (Belkacem et al., 2017).

Diets rich in inulin and SCFAs improve mice lung pathology after infection with IFV by promoting the differentiation of alternatively activated macrophages (AAMs) from circulating Ly6C- monocytes and decreasing the immunopathological effects of neutrophils. Also SCFAs increases anti-IFV immunity by enhancing the CD8^+^ T cells activity by serving as a substrate for fatty acid oxidation and by specifically interacting with the receptor GPR41 (Trompette et al., 2018). One study showed that activation of GPR43 and interferon-α/β receptor (IFNAR) in pulmonary epithelial cells by SCFA acetate induced increased levels of IFN-β in the lungs and increased protection of mice in an experimental model of RSV infection (Antunes et al., 2019).

Other probiotic-derived metabolites also require further investigations. A peptide P18 produced by the probiotic *Bacillus subtilis* 3 (UCM B-5007) share high structural homology with IFV neutralizing antibody, and it is capable of inhibit IFV replication *in vitro* and protect 80% of mice from lethal IFV infection when administered in a therapeutic regimen. This protection is superior to that observed using the anti-viral drug oseltamivir (approximately 70%) (Starosila et al., 2017).

Double-stranded RNA intermediates from IFV and RSV are associated with changes in the host’s coagulation process by activation of receptors such as TLR-3, and retinoic acid-inducible gene I (RIG-I). The activation of these receptors by these viruses, increases the expression of coagulation factors in endothelial cells and monocytes and inhibits fibrinolysis, inducing a prothrombotic state in the hosts, leading to fibrin deposition in the pulmonary alveoli and exacerbation of tissue inflammation. In order to address this issue one study demonstrated in a murine model of IFV and RSV infection, that oral administration of *L. rhamnosus* CRL 1505 in mice increases the clearance of both viruses and controls immune-coagulative responses initiated by the activation of TLR-3 in the lungs, in a process dependent on IL-10 (Zelaya et al., 2014).

Intranasal administration of viable or heat-killed *L. plantarum* NCIMB 8826 and *L. reuteri* F275 protected mice from lethal pneumonia virus of mice (PVM) infection. The lungs showed minimal inflammation, with fewer granulocytes and an increased number of lymphocytes, correlated with a reduction in proinflammatory cytokines CXCL10, CXCL1, CCL2, and TNF-α. Evaluation of the lymphocyte populations demonstrated that treatment did not result in changes in the relative proportions of CD4^+^ T cells (CD3^+^CD4^+^CD8^−^), CD8^+^ (CD3^+^CD4^−^CD8^+^), or B cells (B220^+^). In contrast, the fraction of NK cells (CD3^−^DX5^+^) decreased. The authors demonstrated that these results are not specific for *L. plantarum* NCIMB 8826 and *L. reuteri* F275, as the same protection was observed when using the non-pathogenic gram-positive bacteria *Listeria innocua*. These probiotics also increased the survival of mice infected with the PVM, with the deleted MyD88 (TLRs adapter protein) gene (MyD88^−/−^), thus demonstrating that this induced protection can be TLR-independent (Gabryszewski et al., 2011). However, other studies have determined that the anti-viral activity of probiotics is related to the activation of TLRs. This was demonstrated in mice with *L. rhamnosus* probiotics CRL1505 and CRL1506 that differentially activate the TLR3/RIF-I pathway to inoculate with poly (I:C) (a molecular pattern associated with viruses). The activation of TLR3/RIF-I leads to increased production of IFN-γ, IFN-β, TNF-α, IL-6, and IL-10, the frequency of CD3^+^CD4^+^IFN-γ^+^, CD3^+^CD4^+^IL-10^+^, and the dendritic cells D11c^+^CD11b^low^CD103^+^ and CD11c^+^CD11b^high^CD103, in the lungs. Additionally, this study showed an increase in MHC-II levels in both populations of dendritic cells. The authors also demonstrated that this protective response and modulation of the immune response were similar to those observed in mice infected with the human RSV strain (Tomosada et al., 2013).

Clinical studies showed that probiotics have general effects on viral infections of the respiratory tract (Table 2). A randomized, double-blind, placebo-controlled study, on preterm infants showed that a synbiotic composed of *L. rhamnosus* GG ATCC5310 and galactooligosaccharides and polydextrose reduced the rate of rhinovirus infection compared to the placebo group (Luoto et al., 2014). In school-aged children, consumption of *L. brevis* KB290 during the influenza season was associated with a reduction in the clinical diagnosis of IFV infection (Waki et al., 2014). In a randomized, double-blind, placebo-controlled pilot study, the probiotic *L. rhamnosus* GG was also associated with a reduction in the occurrence of influenza infections (Wang et al., 2018).

As demonstrated, the most used probiotics in studies of pulmonary diseases caused by viruses are also those of the *Lactobacillus* genus. The effects of this genus related to increased protection against viruses are linked to increased production of IFN types I and II, proinflammatory cytokines such as IL-12 and IFN-γ, or even increased expression of genes encoding anti-viral factors. Unfortunately, a mechanistic basis for the observed beneficial effects of probiotics in combating viral lung infections is often not well defined. This knowledge gap is mainly because most experiments using probiotics for viral treatment use different study designs and experimental models, doses, times, and routes of administration. Therefore, more research is needed to understand better the role of probiotics in our immune system in fighting viral pulmonary infections.

#### The Gut-Lung Axis During SARS-CoV-2 Infection

The severe acute respiratory syndrome coronavirus 2 (SARS-CIoV-2), which causes coronavirus disease 2019 (COVID-19), has spread around the world since 2019 and has been declared a pandemic that continues to spread with devastating consequences to public health. As of July 2021, there were approximately 190, 600, 300 global confirmed cases and 4,130,000 confirmed deaths.

SARS-CoV-2 can invade human cells by binding its spike protein to a variety of receptors, such as angiotensin-converting enzyme 2 (ACE2), neuropilin-1, tyrosine-protein kinase receptor (AXL), and antibody–FcγR complexes (V’kovski et al., 2021). The current evidence suggests that the severity of COVID-19 is a consequence of a hyperinflammatory immune response culminating in a ‘cytokine storm’, with markedly increased levels of proinflammatory cytokines such as IL-1, IL-6, IL-12, IFN-γ, and TNF-α, which elicit extensive local and systemic tissue damage (Coperchini et al., 2020).

Recent studies have revealed that patients infected with SARS-CoV-2 demonstrate intestinal dysbiosis, which correlates with the susceptibility and severity of COVID-19 (Zuo et al., 2020; Yeoh et al., 2021). Though some studies detected SARS-CoV-2 RNA in the feces of patients, the activity and infectivity of SARS-CoV-2 in the GI tract are still largely unknown (Zuo et al., 2021). However, as ACE2 is highly expressed in the intestinal epithelia, this receptor may be involved in gastrointestinal symptoms that are common in severe cases (Chen et al., 2020; Villapol, 2020; Koester et al., 2021). Interestingly, in a study with gnotobiotic rats, researchers demonstrated that the gut microbiota regulates the colonic mRNA of ACE2 (Yang et al., 2020).

Another study demonstrated that patients with severe COVID-19 had a significant decrease in the abundance of butyrate-producing bacteria, such as *Faecalibacterium prausnitzii*, *Clostridium butyricum*, *Clostridium leptum*, and *Eubacterium rectale,* and an increased number of common opportunistic pathogens, *Enterococcus* and *Enterobacteriaceae* (Tang et al., 2020)*.* In non-human primates infected with SARS-CoV-2, 16S rRNA analysis of the microbial gut community showed changes in the taxonomic composition, with the relative abundance of *Acinetobacter* and *Ruminococcaceae* being positively correlated with the presence of SARS-CoV-2 in the URT. In addition, SARS-CoV-2 infection significantly alters the metabolite composition with a reduction in the levels of SCFAs, bile acids, and tryptophan metabolites (Sokol et al., 2021).

The impact of probiotics in COVID-19 and in the cytokine storm can be deduced by their known mechanisms in modulating immune response and inflammation (He et al., 2020; de Oliveira et al., 2021), but more basic and clinical research is needed to show their benefits. In a retrospective study of ICU patients diagnosed with pneumonia caused by SARS-CoV-2, the association of the best available therapy with the probiotics *S. thermophilus* DSM 32245, *B. lactis* DSM 32246, *B. lactis* DSM 32247, *L. acidophilus* DSM 32241, *L. helveticus* DSM 32242, *L. paracasei* DSM 32243, *L. plantarum* DSM 32244, and *L. brevis* DSM 27961 showed a positive association with reduced mortality (Ceccarelli et al., 2021).

### Gut-Lung Axis Modulation in the Context of Fungal Lung Infections

Among the wide variety of respiratory pathogens, fungi are responsible for only a small proportion of nosocomial or community-acquired pneumonia. However, these species are of relevant medical interest, as fungi cause high morbidity and mortality especially when they affect immunosuppressed patients, or patients with chronic lung diseases, such as chronic obstructive pulmonary disease (COPD) (Charlson et al., 2012; Delhaes et al., 2012).

Although some studies showed that the microbiota in the gut-lung axis is fundamental to the host response to lung infections by fungi, most studies that have demonstrated antimycotic action against respiratory pathogens have been carried out *in vitro*. *In vitro* experiments showed that the bacterium *Bacillus safensis* can block, in a contact-dependent manner, several *Cryptococcus neoformans* virulence factors including melanin, antiphagocytic capsule, and biofilm formation (Mayer and Kronstad, 2017). Many microorganisms of the genus *Bacillus* are characterized as probiotics (Hong et al., 2005); however, *B. safensis* has not yet been characterized as such. *B. safensis* is phylogenetically close to the probiotic *B. pumilus* (Satomi et al., 2006), and its anti-fungal activity has been described (Pandya and Saraf, 2015), making *B. safensis* an exciting candidate for studies of biological safety and probiotic activity. Another study demonstrated *in vitro* that treatment with concentrated cell-free supernatant from the culture of *L. plantarum* 16 altered the transcription of genes involved in a variety of cellular functions, especially those related to cellular metabolism, which culminated in the complete inhibition of spore germination and development of the germ tubes and hyphae of the pathogen *A. fumigatus* (Crowley et al., 2013). In one *in vivo* study using the probiotics *Saccharomyces boulardii*, *L. paracasei* ST-11, and *L. rhamnosus* GG, mice were not protected against lung infection caused by the pathogen *Cryptococcus gattii* (Oliveira et al., 2017).

The recognition of lectins in the fungal cell wall by PRRs is crucial for the activation of dendritic cells and macrophages and the activation of T cells, including Th1 and Th17, which are the best defense strategies against fungal infections, as they help promote the clearance of fungi through innate effectors such as neutrophils and macrophages. The activation of Treg cells and anti-inflammatory cytokines is also fundamental to the anti-fungal immune response, as these cells and molecules are essential for controlling the inflammatory response. The immune response against fungi has already been extensively reviewed by other authors, such as Lionakis et al. (2017).

Among the opportunistic species that affect the lungs, *Aspergillus* spp. are the primary etiologic agents of invasive lung diseases and mainly affect transplant patients (Kontoyiennis et al., 2010; Pappas et al., 2010). Evidence also suggests that COPD patients are at a high risk of developing invasive aspergillosis, although this association is poorly explored (Hammond et al., 2020). An experimental study demonstrated the importance of intestinal microbiota in structuring the pulmonary anti-*Aspergillus* immune response. During infection by *A. fumigatus*, the administration of antibiotics decreased the number of Th17 cells and IL-17 in the lungs, which correlated with a decrease in intestinal colonization by segmented filamentous bacteria (SFB). By investigating how commensal SFBs were linked to this phenotype, the authors, through serum transfer experiments from mice colonized by SFB to negative SFB mice, demonstrated that SFBs contribute to the accumulation of Th17 cells in the lung by inducing an increase in IL-1. This was confirmed when mice that received serum pre-incubated with an IL-1 antagonist attenuated the response of Th17 cells in the lungs (McAleer et al., 2016). Germ-free mice (GF) infected with *C. gattii* showed greater susceptibility to lung colonization, mortality, correlated with reduced levels of IFN-γ, IL-1β, and IL-17 and reduced phagocytosis and ROS production than conventional mice. After restoring the intestinal microbiota those mice mounted a stronger response to infection by *C. gattii*, associated with prolonged survival rates and higher levels of inflammatory mediators (Costa et al., 2016).

*Pneumocystis jirovecii* is another opportunistic fungus that causes pneumonia, particularly in HIV-positive patients, with an inverse relationship between the CD4^+^ T cell count in the blood and the risk of infection by *P. jirovecii* (Dunbar et al., 2020). When investigating the diversity of the intestinal microbial community between immunocompetent mice and mice depleted of CD4^+^ T cells, with pneumonia caused by *Pneumocystis murina*, there was a significant change in alpha and beta diversity and a change in the taxonomic abundance of the intestinal microbiota among these groups, suggesting that the loss of CD4^+^ T cells affects the intestinal microbiota and the response to *P. murina*. *P. murina* infection was also found to increase the expression of genes in the intestinal microbiota related to carbohydrate energy metabolism, xenobiotic degradation, and signal transduction pathways (Samuelson et al., 2016a).

Another study demonstrated that vaccination, using a prime-boost vaccination strategy, with live *P. murina* induced protection against subsequent lung infection with the same pathogen in immunocompetent mice and even in mice depleted of CD4^+^ T cells. In immunocompetent mice, this immunization increased the number of CD4^+^ T cells, CD8^+^ T cells, CD19^+^ B cells, and CD11b^+^ macrophages in the lungs after a respiratory infection and increased the levels of IgG and IgA specific for *P. murina*. A significant reduction in the lung load of *P. murina* was observed in serum transfer experiments from non-infected and immunized mice to infected mice. The beta diversity of the intestinal microbial community in mice immunized with *P. murina* was also altered, suggesting that the effectiveness of this immunization may be partly related to the modification of the microbiota; however, further studies are needed to determine whether changes in the microbiota participate in the induction of immunological memory in *P. murina* (Samuelson et al., 2016b).

Despite the importance of the intestinal microbiota and its metabolites for the development of anti-fungal immune responses, and *in vitro* studies demonstrate that probiotics have an action against fungi that cause lung infections, there have been no reports of robust studies aiming to assess the effectiveness of the modulation of *in vivo* intestinal microbiota for the prevention and/or treatment of pulmonary fungal infections. Thus, with the significance of lung infections in mind, more researchers urgently need to turn their attention to this broad and promising field.

### Gut-Lung Axis Modulation in the Context of Parasitic Lung Infection

Many helminths cause disease, but they have been shown to also influence the pulmonary immune response. Similar to bacteria, they co-evolved with the host’s immune system to maintain a mutually beneficial relationship (Schwartz et al., 2018). Some helminths also share the same niche, the intestinal lumen, with bacteria belonging to the microbiota, and some studies have shown that there are complex interactions between the two (Leung et al., 2018). The intestinal microbiota acts as one of the main inducers of the activation and function of local and systemic antiparasitic responses, such as the activation of Th2 cells and eosinophils (Jiménez-Saiz et al., 2020).

During the larval phase of their life cycle, different species of helminths, such as *Ascaris lumbricoides*, *Toxocara* sp., *Necator americanus*, *Ancylostoma duodenale,* and *Strongyloides* sp., migrate through the lungs and induce pathological immune responses and cause tissue damage, such as eosinophilic pneumonia, which is characterized mainly by increased infiltration of eosinophils in the lung parenchyma and blood eosinophilia (Akuthota and Weller, 2012).

Some studies involving the modulation of the microbiota in spite of helminth infections have focused only on intestinal pathology and on the parasitological aspects of the infection, such as intestinal parasitic load, release of eggs in the feces, and survival of mice. This was demonstrated experimentally with the probiotic *L. casei* ATCC7469 in infection with *Trichinella spiralis* (Bautista-Garfias et al., 2001), *B. animalis* 04450B against infection by *S. venezuelensis* (Oliveira-Sequeira et al., 2014), and *S. boulardii* in mice infected with *T. canis* (Avilada et al., 2012). However, researchers have already reported the beneficial effect of probiotics in reducing the parasitic burden of larval stages during *T. canis* infection in mice. *In vitro* experiments demonstrated a reduction in the viability of *T. canis* larvae after direct incubation with live cells or cell-free supernatant of the probiotic *Enterococcus faecalis* CECT712. The same study also showed that oral administration of *E. faecalis* CECT712 significantly reduced the number of *T. canis* larvae found in the lungs of these animals (Chiodo et al., 2010). *L. rhamnosus* (Walcher et al., 2018) and *L. acidophilus* ATCC 4356 (Cadore et al., 2020) were also able to reduce the parasitic larval burden of *T. canis* in the lungs of mice, but had no antiparasitic action against the larvae *in vitro*, which indicates the indirect action of these two probiotics on *T. canis*, probably related to the stimulation of the protective immune response. The administration of *S. boulardii* in mice infected with *T. canis* increased the transcription of genes encoding IL-12 and IFNγ, which correlated with a decrease in the number of larvae in the lungs and other tissues (de Avila et al., 2016).

Some probiotics, mainly from the *Lactobacillus* genera, showed action *in vitro* and *in vivo* against helminths that cycle through the lungs. However, differences in the efficacy of species and strains used can be attributed to variability in the experimental models, the probiotic dose, and the administration route. However, data are insufficient to determine the molecular mechanisms by which probiotics act on helminths that cycle through the lungs. Furthermore, further studies on host-microbiota-helminth interaction mechanisms are needed to validate the actions of probiotics in clinical studies with humans.

## Studies Perspectives and Conclusion

Although studies have shown that probiotics, prebiotics, and synbiotics have prophylactic and therapeutic effects against lung infections caused by bacteria, viruses, fungi, and helminths, further studies are needed to better understand the mechanisms of action and molecular pathways involved in these strategies. It is necessary to favor the translational use of gut microbiota modulation strategies as a therapeutic approach to human lung diseases. In addition, since the effect of these strategies is highly linked to the strains of the microorganism and the dose and route of administration, more in-depth investigations should be performed, considering well-defined experimental protocols. Considering the complexity of the microbiota and its interaction with the host, it is also important to determine whether this strategy acts in synergy with the microbiota or has another mechanism involving direct action against the lung pathogen or modulation of the host immune system. In addition, it is important to emphasize the therapeutic window necessary to restore the gut microbiota to re-establish homeostasis after lung infection control.

## Abbreviations

GF, Germ-free; GPCR, G-protein-coupled receptors; ICU, Intensive care units; IFV, Influenza virus; PRR, attern recognition receptors; PVM, Pneumonia virus of mice; ROS, Reactive oxygen species; RSV, Respiratory syncytial virus; SCFA, Short-chain fatty acids; SFB, Segmented filamentous bacteria; URT, Upper respiratory tract; VAP, Ventilator-associated pneumonia.
